# Serum selenium level and gestational diabetes mellitus: a systematic review and meta-analysis

**DOI:** 10.1186/s12937-016-0211-8

**Published:** 2016-10-28

**Authors:** Fei-Juan Kong, Lei-Lei Ma, Shu-Ping Chen, Ge Li, Jia-Qiang Zhou

**Affiliations:** 1 Department of Anesthesiology, Hangzhou First People’s Hospital, Nanjing Medical University, No. 261 Huansha Road, Hangzhou, 310006 People’s Republic of China; 2 Department of Critical Care Medicine, Zhejiang Provincial People’s Hospital, Hangzhou, China; 3 Department of Endocrinology, Sir Run Run Shaw Hospital, School of Medicine, Zhejiang University, 3 East Qingchun Road, Hangzhou, 310016 People’s Republic of China; 4 Department of Cardiology, Shanghai Institute of Cardiovascular Diseases, Zhongshan Hospital, Fudan University, Shanghai, China

**Keywords:** Gestational diabetes mellitus, Pregnancy, Selenium, Meta-analysis

## Abstract

**Background:**

The association between serum selenium level and gestational diabetes mellitus (GDM) is controversial. The aim of our study was to systematically review available literature linking selenium to GDM for a comprehensive understanding of the relationship between serum selenium level and GDM in human.

**Methods:**

PubMed, The Cochrane Library and Medline were searched for studies published up to August 2016. Manual searches of references of the relevant original studies were carried out. Pooled estimates were measured using the fixed or random effect model. Overall effect was reported in a standard mean difference (SMD). All data were analyzed with Review Manager 5.3 and Stata 12.0.

**Results:**

Of 44 references reviewed, seven studies involving 569 patients met our inclusion criteria and contributed to meta-analysis. All the studies were used to evaluate the relationship between serum selenium level and GDM. Selenium level was significantly lower in women with GDM than those without GDM (SMD = −1.17; 95 % CI: −1.98 to −0.35, *P* = 0.005). Subgroup analysis showed that such trend was consistent within the non-Caucasian population (Asia: SMD = −2.82; 95 % CI: −5.21 to −0.43, *P* = 0.02; Africa: SMD = −0.56; 95 % CI: −1.07 to −0.05, *P* = 0.03) and in the third trimester (SMD = −1.78; 95 % CI: −3.04 to −0.52, *P* = 0.006), but not within the Caucasian population (Europe: SMD = −0.6; 95 % CI: −1.98 to 0.78, *P* = 0.39) or in the second trimester (SMD = −0.68; 95 % CI: −1.6 to 0.25, *P* = 0.15).

**Conclusions:**

The available evidences suggested that serum selenium level was lower in women with GDM than those with normal glucose tolerance, especially within the non-Caucasian population and in the third trimester. However, well-designed prospective studies are needed to understand dynamic associations between selenium status and GDM risk.

## Background

Gestational diabetes mellitus (GDM), one of the most common pregnancy complications, is defined as impaired glucose tolerance that begins or is initially recognized during pregnancy. As few pregnant women show the related clinical symptoms, GDM screening has became a routine prenatal project nowadays. During the last few decades, GDM has affected up to 14 % pregnant women depending on different diagnostic criteria and ethnic origin [1]. A UK study reported that 0.4 % white, 1.5 % African, 3.5–7.3 % Asian, 4.4 % Indian and about 1–4 % mixed-origin women were shown to have gestational diabetes [2]. And the recent studies indicated that the prevalence of GDM was found to be ranging from < 5 % in South Korea, South Africa and UK, to < 10 % in Italy, Turkey, United States and Australia, to a prevalence as high as 20 % in Bermuda and Nepal [3, 4]. GDM received increasing attention globally due to its continuous increase in prevalence, particular in developing countries, including China, India and Africa [5]. In addition to increasing maternal incidence of type 2 diabetes mellitus (T2DM) and metabolic syndrome at follow-up, GDM is associated with various adverse acute outcomes and long-term metabolic derangements in offspring, e.g. childhood obesity and T2DM later in life [6–9]. The well-established risk factors for GDM include high maternal age, obesity or maternal overweight status, prior history of GDM, family history of T2DM, being of a particular race/ethnicity (Pakistan or Indian descent) and previous delivery of a macrosomic infant [7, 8]. Recently, it is proposed that a poor selenium status is associated with the incidence of GDM.

Selenium, as an essential trace element for human health, serves as an integral component of several enzymes, including formate dehydrogenase, glutathione peroxidase, selenoprotein P and W and the deiodinases [10]. In addition to its role in enzyme function, selenium is involved in the complex system of defense against oxidative stress through selenium-dependent glutathione peroxidases (GPXs) and other selenoproteins [10, 11]. Selenium deficiency is reported to be associated with a variety of diseases, including DM, heart disease, autoimmune diseases and certain types of cancers [10]. It is indicated that selenium deficiency is widespread in many countries, especially in developing countries [12]. Currently, the antioxidant functions of selenium are gaining more and more attention for its closer associations with DM, including T2DM [13–15] and GDM [16–22]. It has been indicated that oxidative stress is associated with the etiology, pathogenesis, and complications of DM [23]. In this sense, selenium might be benefit for DM. More important, it has been suggested that selenium exhibits insulin-like properties, which may be involved in maintaining normal glucose uptake, regulating cellular glucose utilization, and reducing the severity of insulin resistance [24, 25]. In view of the non-classic function, selenium might be expected to play a protective role against DM. The scientific evidence linking selenium with T2DM is growing large, but data investigating the correlation between selenium status and GDM are controversial. In this regard, some studies reported that women with GDM showed lower concentrations of serum selenium than healthy pregnant women [16–19], while Molnar *et al* showed the contrary [22]. Meanwhile, no significant association between serum selenium and GDM was documented [20, 21].

In order to provide a more comprehensive estimation of the association between selenium level and GDM, we performed a systematic review and meta-analysis on related studies aiming for getting a more persuasive conclusion.

## Methods

Our review followed the Meta-Analyses and Systematic Reviews of Observational Studies (MOOSE) guidelines [26]. The data were presented according to the recommendations of the PRISMA statement [27].

### Search strategy

A comprehensive systematic literature search was conducted in the published databases including PubMed, The Cochrane Library and Medline up to August 2016. The search strategy included key terms that were summarized as follows: “selenium”, “selenium compounds”, “selenium-binding proteins”, “selenate”, “gestational diabetes mellitus”, “GDM”, “diabetes pregnancy”, “insulin gestation”. References from these relevant studies were manually searched.

### Inclusion and exclusion criteria

Studies were considered eligible if they met the following criteria: (1) GDM as outcome and the control were women with normal glucose tolerance (NGT); (2) all participants did not have a previous history of diabetes or present pregnant complications; (3) full-text articles were published in English. Studies were excluded if they were (1) available only as abstracts, review studies, case reports, expert comment, or editor opinion, (2) experimentation on animals or in *vitro*; (3) predefined outcome data required for analyses were lacking.

### Data extraction and quality evaluation

Two reviewers (FJ Kong and LL Ma) independently reviewed all searched studies and extracted data using a predefined form. If there was a discrepancy, a discussion was carried out to reach an agreement. If a consensus could not be reached, a third experienced investigator (JQ Zhou) was consulted. The following information of each study was recorded: first author, year of publication, country of the study, study design, assay method of selenium, diagnosis criteria of GDM, sample size of the case and control group, Mean and Standard Deviation (SD) (part of the data were converted) of selenium level, trimester of selenium level measurement, and group mean of age and body mass index (BMI) of GDM women.

The individual study quality was assessed according to the Cochrane collaboration’s tool for risk of bias, which contains random sequence generation, allocation concealment, blindness, incomplete outcome data, selective outcome reporting, and other biases.

### Statistical analysis

Standard mean difference (SMD) and 95 % confidence interval (95 % CI) were calculated to assess the differences in serum selenium between groups. Significance levels were determined by Z test. Forest plots were used to demonstrate effect sizes and their CI. Heterogeneity amongst the included studies was assessed by Cochran’s Q statistics and I^2^ statistics. According to heterogeneity inspection results, corresponding pooled method was chosen: if I^2^ > 50 %, random effect model was used; while I^2^ ≤ 50 %, fixed effect model was adapted. We also did subgroup analyses to explore the potential source of heterogeneity if heterogeneity across studies was statistically significant. Potential publication bias was evaluated using Begg’s test and Egger’s test. Sensitivity analyses were carried out by sequentially omitting one single study each time to test the robustness of uncertainty in the meta-analysis. All data were analyzed with Review Manager (RevMan 5.3) statistical sofware provided by The Cochrane Collaboration and Stata 12.0 (Stata Corp, College Station, TX, USA). The significance level was set as 0.05, except Cochran’s Q test for heterogeneity as 0.1.

## Results

### Literature search

**Fig. 1:**
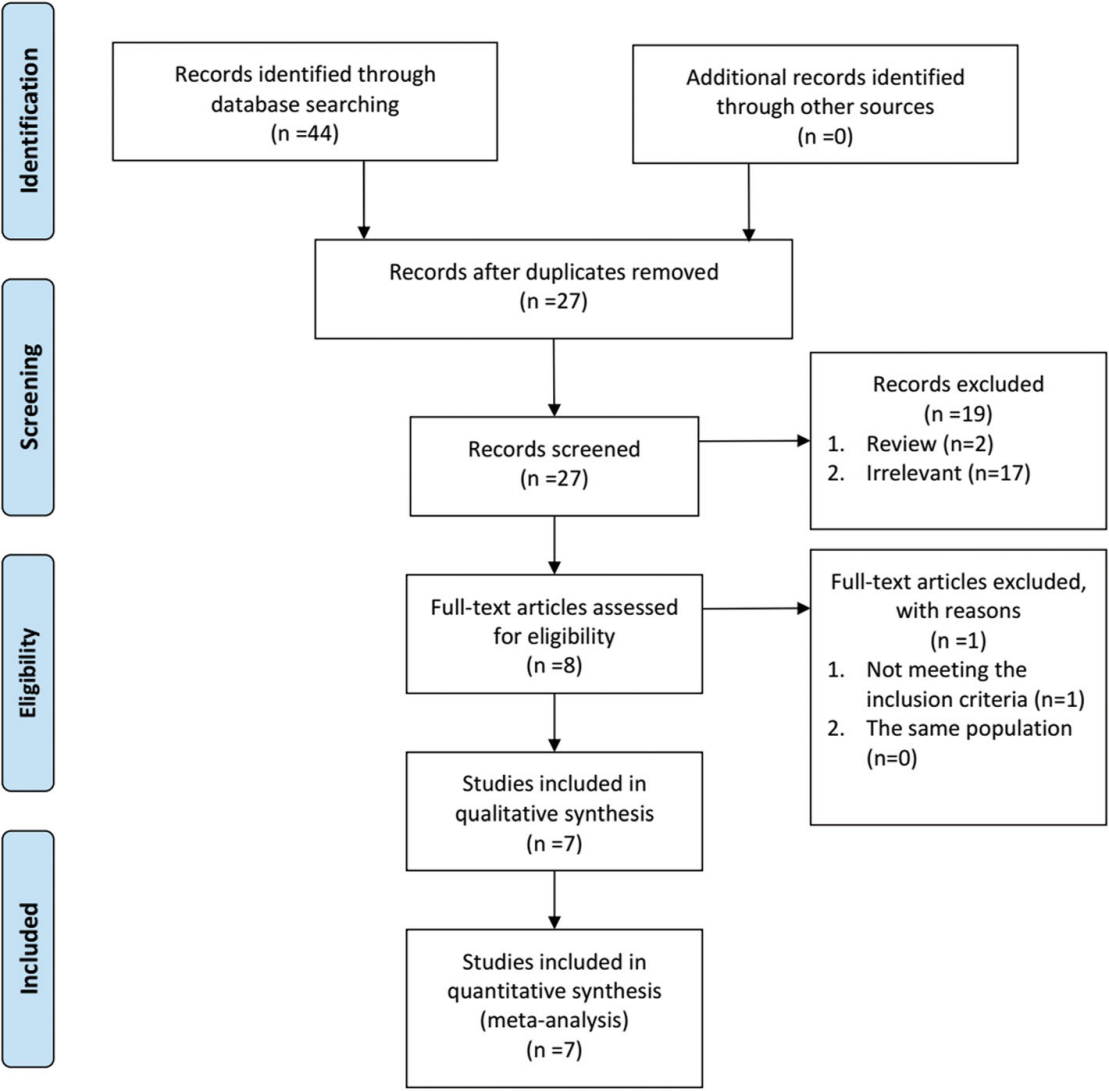
Flow diagram of study recruiting

A flow diagram of the included and excluded studies was shown in Fig. 1. According to the search strategy, 44 citations were identified from the three databases. After removing the duplicates (*n* = 17), two reviewers screened the titles and abstracts of potentially relevant studies (*n* = 27) independently. Finally, a total of seven studies were included for meta-analysis [16–22].

### Study characteristics

All included studies published from 2001 to 2014 were observational studies including 178 GDM patients and 391 healthy pregnant women. The characteristics of the studies included in the present meta-analysis are given in Table 1. Of the seven studies included in the meta-analysis, two were carried out in Kuwait [17, 20], one in China [16], one in Italy [18], one in Turkey [19], one in Hungar [22] and one in Sudan [21]. GDM was usually diagnosed during the 24–28 weeks of gestation. The definition used for GDM was the criteria proposed by Carpenter and Coustan in three studies [18, 19, 21], and the World Health Organization (WHO) criteria in two studies [20, 22]. Blood samples for selenium measurement were collected in the second [16, 18, 19, 22] and third [16, 17, 20, 21] trimester of gestation. Six studies were case-control [16–18, 20–22], and one cross-sectional design [19]. The sample size of these studies ranged from 10 to 123. Three of the included studies involved in the GDM women with BMI < 28 kg/m^2^ [18, 21, 22], and two with BMI ≥ 28 kg/m^2^ [17, 20].

**Table 1:** Characteristics of studies included in the meta-analysis

Study	Location	Study type	Sample	Methods	GDM criteria	Case group		Control group		Selenium measurement trimester	Average age	Average BMI
						Sample size	Selenium	Sample size	Selenium			(kg/m^2^)
Tan, 2001 [16]	China	Case-control	Serum	AFS	N/A	57	66 ± 12	40	78.5 ± 17.7	Second	N/A	N/A
		Case-control	Serum	AFS	N/A	83	61.5 ± 13.1	50	70.7 ± 15.2	Third	N/A	N/A
Al-Saleh, 2004 [17]	Kuwait	Case-control	Serum	AAS	N/A	15	75.2 ± 3.1	15	102.3 ± 3.1	Third	31	28.57
Bo, 2005 [18]	Italy	Case-control	Serum	AAS	C & C	29	8.8 ± 1.3	123	10.8 ± 1.8	Second	33.5	25.4
Al-Saleh, 2007 [20]	Kuwait	Case-control	Serum	AAS	WHO	10	85.1 ± 5.4	11	89 ± 4.9	Third	32	36.26
Kilinc, 2008 [19]	Turkey	Cross-sectional	Serum	AAS	C & C	30	34.7 ± 8.7	101	50.7 ± 9.8	Second	25	N/A
Molnar, 2008 [22]	Hungary	Case-control	Serum	AAS	WHO	17	51.7 ± 11.62	20	40.5 ± 8.03	Second	31	24.1
Hamdan 2014 [21]	Sudan	Case-control	Serum	AAS	C & C	31	164.4 ± 59.0	31	204 ± 78.83	Third	32	25.8

*N/A* not available or not reported, *AFS* atomic fluorescence spectrometric, *AAS* atomic absorption spectrometry, *GDM* gestational diabetes mellitus, *C&C* carpenter and coustan, *WHO* World Health Organization, *BMI* body mass index

**Fig. 2:**
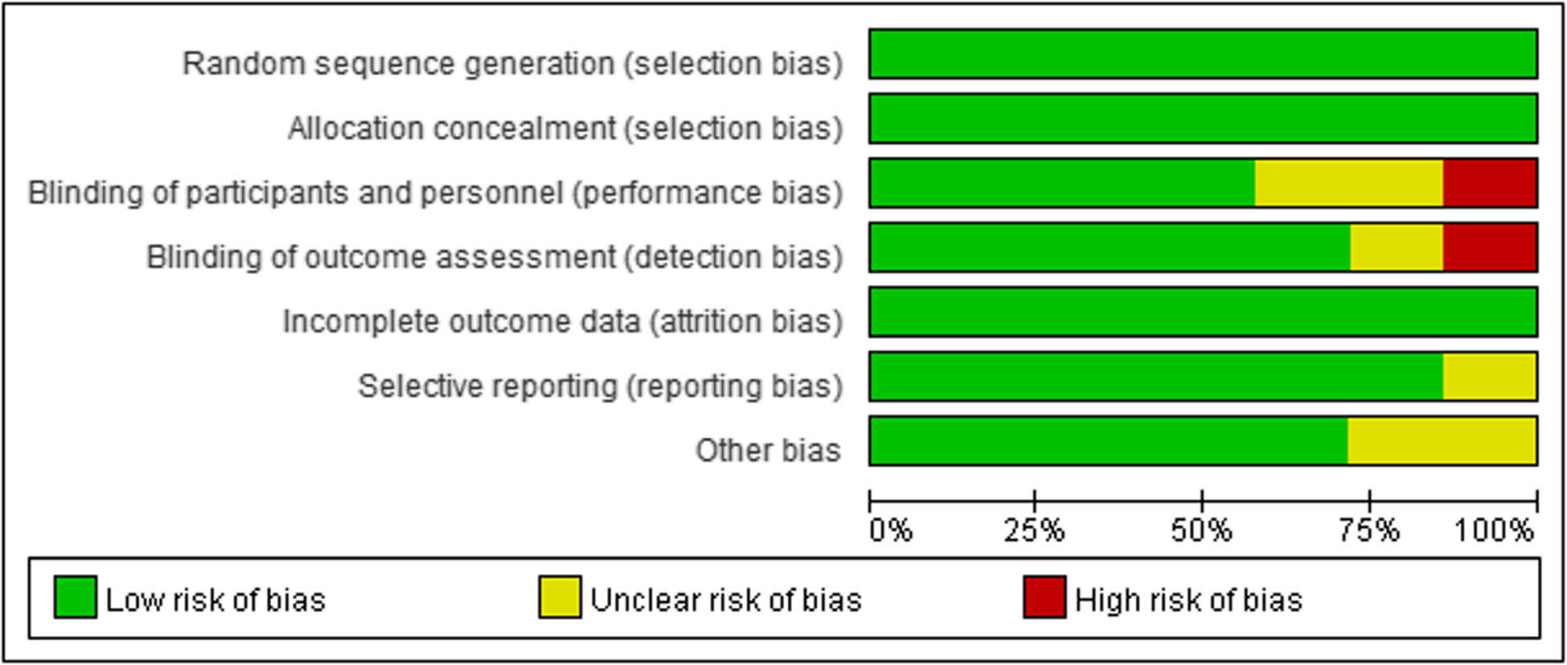
Risk of bias graph. The Cochrane collaboration’s tool was used to evaluate risk of bias

The assessment on the quality of the included studies was demonstrated in Fig. 2.

### Overall meta-analysis

**Fig. 3:**
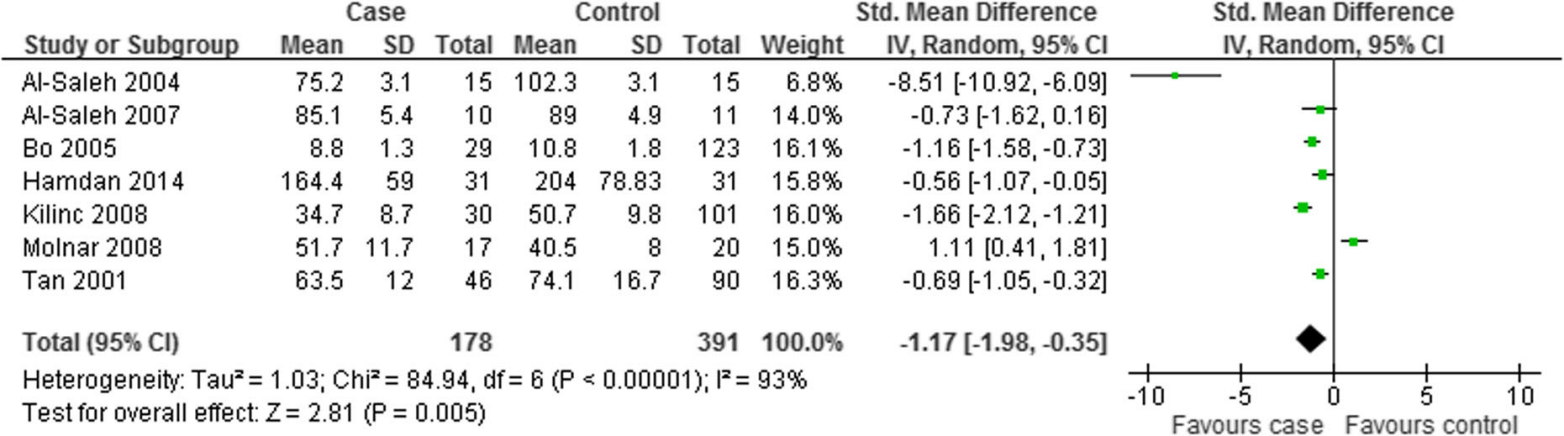
Forest plot of the serum selenium level in GDM or healthy pregnant women. The random effect model (Inverse Variance method) was applied

As indicated in Fig. 3, the overall level of serum selenium in GDM patients was lower than that in the healthy controls with statistical significance (SMD = −1.17; 95 % CI: −1.98 to −0.35, *P* = 0.005). The SMDs from the individual studies were analyzed using random-effects models, as the heterogeneity was considered significant (*P* < 0.00001, I^2^ = 93 %).

**Fig. 4:**
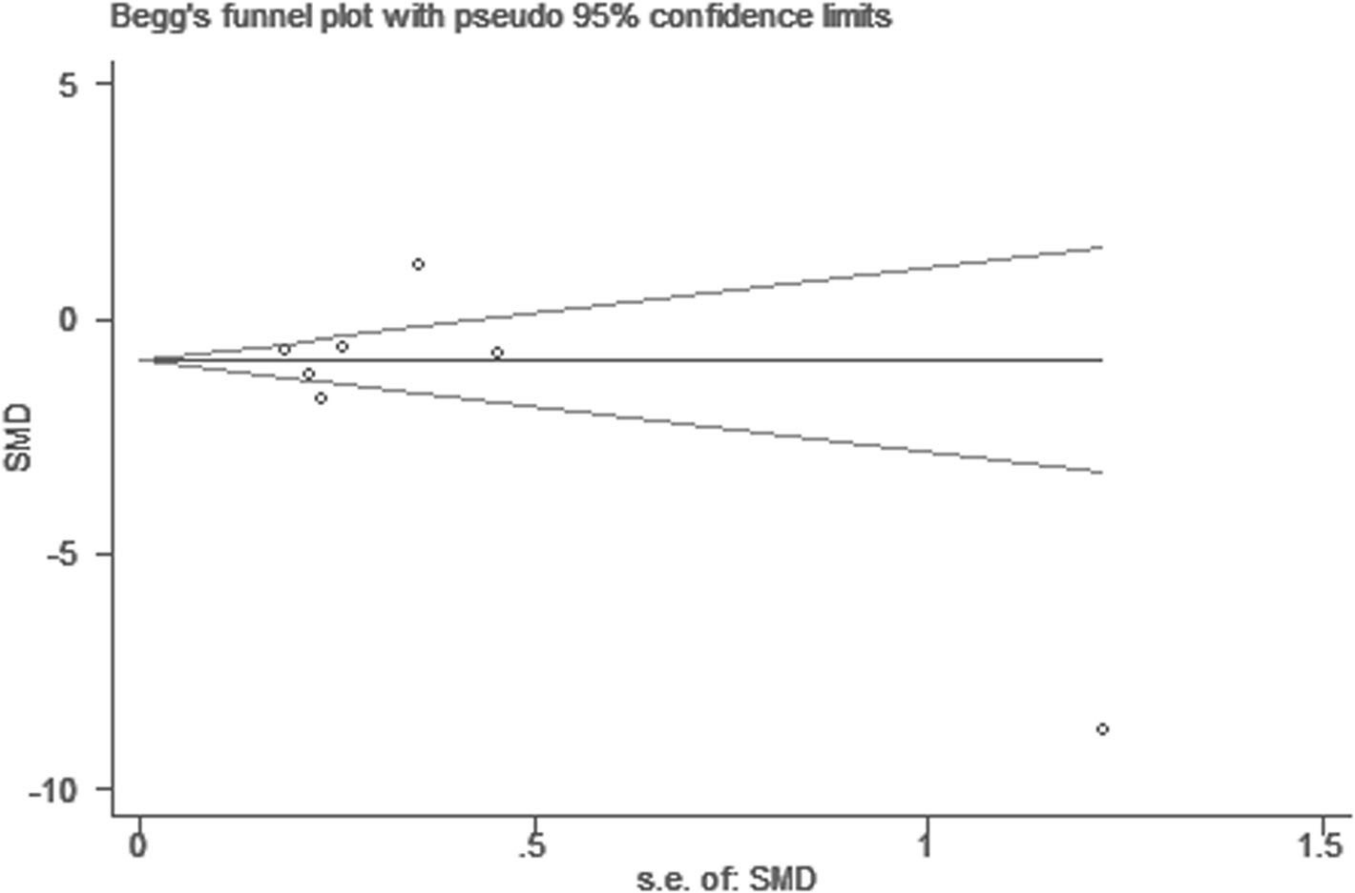
Begg’s funnel plot of included studies for potential publication bias

**Fig. 5:**
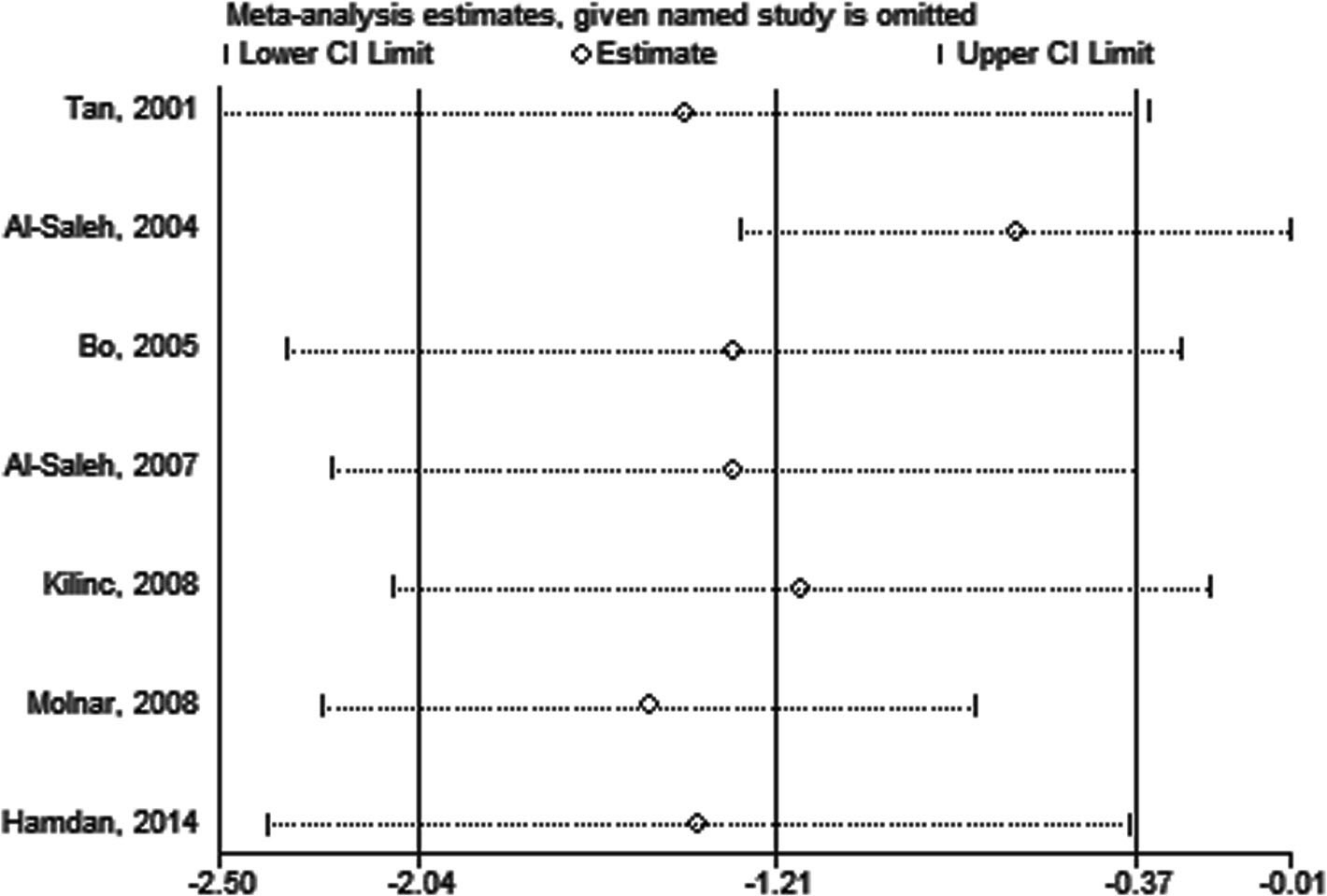
Sensitivity analysis of the serum selenium level in GDM or healthy pregnant women

**Fig. 6:**
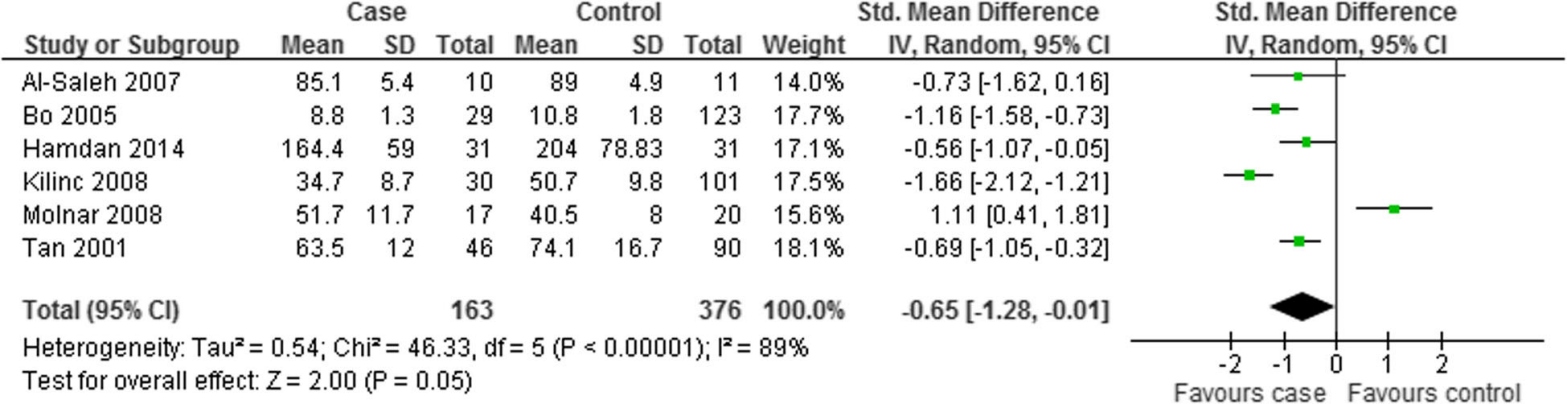
Forest plot of the serum selenium level in GDM or healthy pregnant women after the extraction of one study. The random effect model (Inverse Variance method) was applied

No significant publication bias was found in our meta analysis as indicated in Fig. 4 (Begg’s test: *P* = 0.76; Egger’ test: *P* = 0.5). Sensitivity analysis was carried out by omitting studies one by one to explore potential sources of heterogeneity and assess relevant changes on the combined results. As suggested in Figs. 5 and 6, the estimates effects showed material alteration when inclusion or exclusion of the study conducted by Al-Saleh *et al* [20], ranging the estimates effects from −1.98 to −0.35 (*P* = 0.005) for inclusion, and from −1.28 to −0.01 (*P* = 0.05) for exclusion. However, significant decrease in the heterogeneity was not detected (inclusion: *P* < 0.00001, I^2^ = 93 %; exclusion: *P* < 0.00001, I^2^ = 89 %). After strict screening again, we found that the study did meet the exclusion criteria. Thus, the findings were robust against study deletions.

### Subgroup analysis

**Fig. 7:**
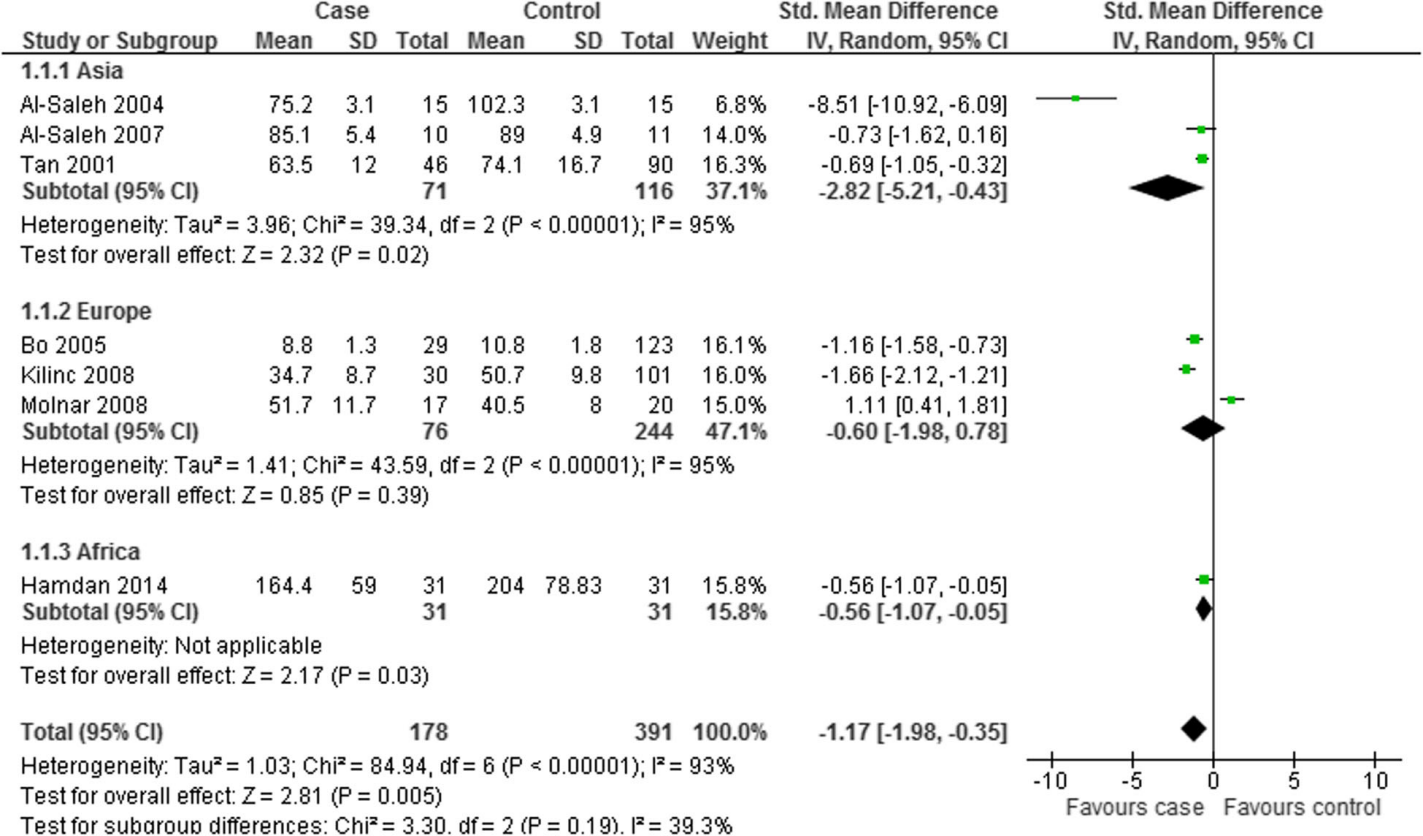
Subgroup analysis of serum selenium level in GDM or healthy pregnant women based on geographic location. The random effect model (Inverse Variance method) was applied

**Fig. 8:**
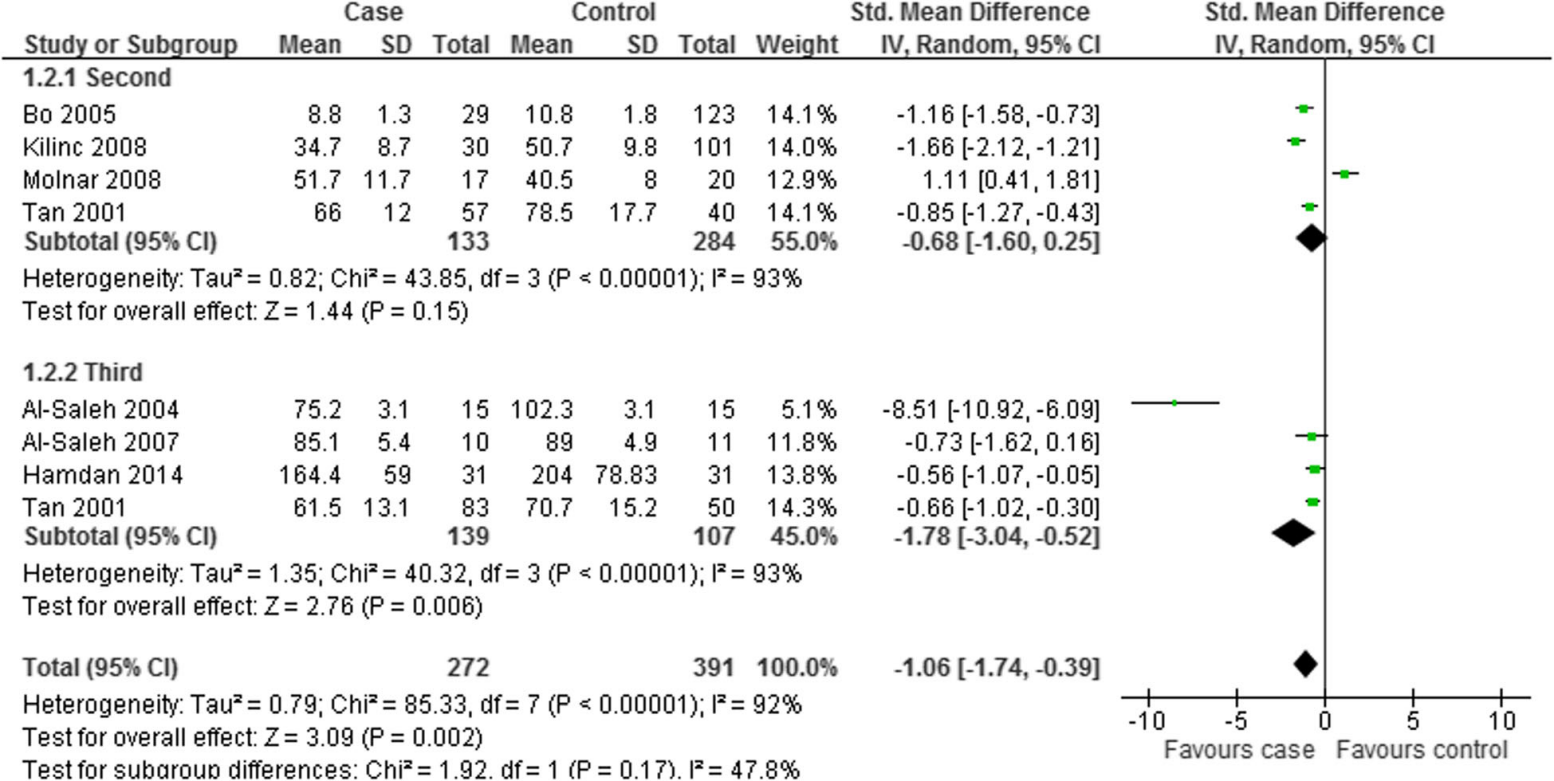
Subgroup analysis of serum selenium level in GDM or healthy pregnant women based on different trimester. The random effect model (Inverse Variance method) was applied

To explore the source of the heterogeneity and obtain thorough information from this meta-analysis, subgroup analysis was further carried out. Subgroup analysis was carried out by geographic site and the trimester of selenium measurement. The comprehensive results were shown in Figs. 7 and 8.

When stratifying by geographic site, these studies were classified as the Asian group [16, 17, 20], European group [18, 19, 22], and African group [21]. The three papers carried out in Asia and Europe respectively did present a conclusion of heterogeneity (Asia: *P* < 0.00001, I^2^ = 95 %; Europe: *P* < 0.00001, I^2^ = 95 %), and therefore the random effect model was chosen to do pooled analysis. The results in Figs. 7 revealed that the serum level of selenium in the non-Caucasian population was decreased in GDM women (Asia: SMD = −2.82; 95 % CI: −5.21 to −0.43, *P* = 0.02; Africa: SMD = −0.56; 95 % CI: −1.07 to −0.05, *P* = 0.03). However, the index of selenium level in Caucasians’ serum demonstrated no statistical significance (Europe: SMD = −0.6; 95 % CI: −1.98 to 0.78, *P* = 0.39).

In the subgroup analysis depending on the trimester of selenium measurement, the random effect model was chosen to do pooled analysis because significant heterogeneity was observed (Second trimester: *P* < 0.00001, I^2^ = 93 %; Third trimester: *P* < 0.00001, I^2^ = 93 %). As demonstrated in Figs. 8, the difference of selenium level between the GDM group and controls was significant in the subgroup analysis of the third trimester (SMD = −1.78; 95 % CI: −3.04 to −0.52, *P* = 0.006), while such trend was not detected in the subgroup analysis of the second trimester (SMD = −0.68; 95 % CI: −1.6 to 0.25, *P* = 0.15). We did not perform subgroup analysis according to BMI and average age because of insufficient data in some studies.

## Discussion

This systematic review and meta-analysis found that serum level of selenium was significantly lower in women with GDM than healthy pregnant controls. The conclusion was especially available in the subgroups of the non-Caucasian population and the third trimester. The changes of selenium level in GDM patients had been already suggested by other authors, and a meta-analysis was made in 2015 [28], which indicated a significant decrease of serum selenium concentration in pregnant women with gestational hyperglycemia from six observational studies. Similarly, our meta-analysis of seven independent observational studies provided strong evidence that serum selenium level in women with GDM are significantly lower than the healthy pregnant controls. More importantly, compared with the previous meta-analysis [28], our meta-analysis conducted more comprehensive and thorough analysis and proposed more convincing and detailed results.

GDM is defined as any degree impairment of glucose intolerance with onset or first recognition during pregnancy. Pregnancy is typically accompanied by physiological insulin resistance that begins the second trimester and progresses through the third trimester, leading to an increase in maternal insulin secretion to maintain blood glucose levels as a consequence of adaptive pancreatic beta-cell proliferation. Exacerbation of pancreatic beta-cell dysfunction or impairment of compensatory increases in insulin secretion from these cells or both results in GDM [29]. In view of the prevalence of GDM, an increasing number of studies have involved in exploring the physiological and pathological mechanisms of GDM in animal models and human beings [30–35].

In recent years, a growing advancement in understanding the biochemical mechanisms and cellular targets of trace minerals has rekindled the interests of researchers towards their role in DM. Selenium is an essential trace element and currently, several studies have investigated the role of selenium in human diseases, particularly in the context of DM [36, 37]. Previous studies focuses on the relationship between selenium and T2DM or obesity, and the focus shifts to GDM recently. However, the evidence about the relationship between selenium and GDM is conflicting. Some studies claimed that patients with GDM showed significantly decreased selenium concentrations compared with healthy subjects [16–19]. Tan *et al* revealed that serum selenium level decreased to lower if pregnant women suffered from impaired glucose tolerance (IGT) or GDM and indicated an inverse correlation between gestational period and serum selenium levels [16]. The results were convinced by Bo, Al-Saleh, and Molnar study group [17–19]. Pregnancy-associated factors including hemodilution of pregnancy and increased requirement for fetal growth may attribute to some of the observed decrease in the serum selenium concentration [38, 39]. In addition, increase of insulin resistance occur in parallel with increasing in oxidative stress during pregnancy [40], which is more prominent in the condition of GDM. Selenium is believed to be one of the most important antioxidant nutrients in the human body, and selenoproteins have a protective effect against oxidative stress and inflammation [11, 25]. In the state of GDM characterized by chronic hyperglycemia or insulin resistance, individuals are more susceptible to various stimuli-mediated oxidative stress, which may lead to the overconsumption of antioxidants, resulting in the decrease of selenium [41]. On the contrary, in view of insulin-like properties of selenium [25], a poor selenium status may aggravate insulin resistance induced by pregnancy, leading to GDM. Therefore, it is not yet known whether low selenium status is contribute to disease etiology, or may be a consequence of disease that aggravates the condition further [10].

Conversely, some studies reported that patients with GDM showed increased selenium concentrations compared with control subjects [22]. The potential mechenisms are not clear because of the insufficient studies. Meanwhile, Hamdan group suggested that there was no significant correlations between selenium and GDM or between selenium and BMI or gestational age [21]. The same study gruop Al-Saleh assessed the selenium levels in obese GDM patients (BMI > 30 kg/m^2^) and concluded that serum selenium concentrations and antioxidant enzyme status were not associated with obese GDM [20], which might be due to the difference in BMI of GDM and the corresponding controls.

The current meta-analysis showed that the pooled value of Mean [95 % CI] was of statistical significance, revealing decreased serum levels of selenium in GDM women. However, according to the over all forest plot in Fig. 3, substantial heterogeneity (I^2^ = 93 %) was observed among the studies. To find the source of heterogeneity, subgroup analysis and sensitivity analysis were carried out. Subgroup analysis was depended on geographic site and the trimester of selenium measurement. In the subgroup analysis, the overall circulating selenium level of GDM patients was relatively lower than that in healthy pregnant women within the non-Caucasian participants (Asia and Africa) and in the third trimester. However, the trend was not available in the subgroup analysis of Caucasian patients (Europe) and in the second trimester. The dietary habits in different locations and higher tendency of insulin resistance and increasing level of oxidative stress in the third trimester may contribute to the above results. Diet is the major source of selenium intake for the general population. Dietary selenium is mainly derived from animal protein, e.g. muscle meats, organ meats, and seafood. And the content of selenium in grains and seeds depends on the soils in which they are grown. Europeans take meat, milk and wheat as their staple food, which are rich in selenium, and pregnancy does not change their dietary habits too much; while Asians and Africans rely mainly on vegetables and grains, which can not provide adequate selenium, and an appropriate increase in the intake of meat products may be suggested during pregnancy. The difference in dietary habits may explain the different results in the African and Asian population and European group. In view of insufficient data of BMI and average age in some studies, other subgroup analysis was not performed. When the subgroup analysis of different countries and measurement time was carried out, we found that the heterogeneity was not removed. Additionally, the sensitivity analysis was performed to show relevant changes on the combined results by study removals. The results indicated that the final meta-analysis results were not stable enough with inclusion or exclusion of the study conducted by Al-Saleh *et al* [20], which may probably contribute to the heterogeneity. However, the heterogeneity was still existed after exclusion of the mentioned study. The relevant clinical data did not provided by the studies and methodological difference may attribute to the heterogeneity observed in our meta-analysis.

Giving the acute adverse outcomes and long-term effects on pregnant women and offspring, GDM is drawing increasing attention in recent years. The topic is therefore of relevance from the public health perspective and the present meta-analysis can contribute to clarify some of pathophysiological mechanisms of GDM by providing statistical assessment. However, there were some limitations to this meta-analysis. First, the publication bias cannot be avoided absolutely, as only published studies in English in the selected databases were included. Second, the measurement methods of selenium were not consistent and the absolute value of selenium was widely different among included studies. Third, different diagnostic criteria of GDM may influence the pooled effect due to different threshold value for oral glucose tolerance test. Fourth, we have no access to get the original data of the included literature, so we cannot guarantee the accuracy of the data. Fifth, some of the studies did not provide enough clinical information, we can not perform thorough and further analysis to explore the source of heterogeneity. Therefore, the results should be interpreted with caution.

## Conclusion

In conclusion, the meta-analysis revealed that selenium level was lower in patients with GDM. Women with GDM generally have few obvious related symptoms, then selenium level might be a potential risk factor for assessing GDM. This result could help clinical staff to instruct women with GDM to prevent the progression of GDM. However, the regulation and metabolism of selenium remained unclear in human. For further studies, well-designed epidemiological studies with large sample sizes and strict stratification of potential confounding factors should be performed. It will be meaningful and interesting to explore the potential role of selenium in GDM prediction and therapeutics.

## Abbreviations

95 % CI: 95 % confidence interval
BMI: Body mass index
GDM: Gestational diabetes mellitus
GPXs: Glutathione peroxidases
IGT: Impaired glucose tolerance
MOOSE: Meta-analyses and systematic reviews of observational studies
NGT: Normal glucose tolerance
SD: Standard deviation
SMD: Standard mean difference
T2DM: Type 2 diabetes mellitus
WHO: World Health Organization
